# Impact of clinicians’ behavior, an educational intervention with mandated blood pressure and the hypotension prediction index software on intraoperative hypotension: a mixed methods study

**DOI:** 10.1007/s10877-023-01097-z

**Published:** 2023-12-19

**Authors:** Ilonka N. de Keijzer, Jaap Jan Vos, David Yates, Caroline Reynolds, Sally Moore, Rebecca J. Lawton, Thomas W.L. Scheeren, Simon J. Davies

**Affiliations:** 1 Department of Anesthesiology, University Medical Center Groningen, University of Groningen, Hanzeplein 1, Groningen, 9700 RB The Netherlands; 2 Department of Anesthesia, Critical Care and Perioperative Medicine York Teaching Hospitals NHS Foundation Trust, Centre for Health and Population Sciences, Hull York Medical School, York, UK; 3 Bradford Institute for Health Research, Bradford Teaching Hospitals Foundation Trust, Bradford, UK; 4 University of Leeds, Leeds, UK

**Keywords:** Blood pressure management, Intraoperative hypotension, Hemodynamic monitoring, Hypotension Prediction Index

## Abstract

**Purpose:**

Intraoperative hypotension (IOH) is associated with adverse outcomes. We therefore explored beliefs regarding IOH and barriers to its treatment. Secondarily, we assessed if an educational intervention and mandated mean arterial pressure (MAP), or the implementation of the Hypotension Prediction Index-software (HPI) were associated with a reduction in IOH.

**Methods:**

Structured interviews (n = 27) and questionnaires (n = 84) were conducted to explore clinicians’ beliefs and barriers to IOH treatment, in addition to usefulness of HPI questionnaires (n = 14). 150 elective major surgical patients who required invasive blood pressure monitoring were included in three cohorts to assess incidence and time-weighted average (TWA) of hypotension (MAP < 65 mmHg). Cohort one received standard care (baseline), the clinicians of cohort two had a training on hypotension and a mandated MAP > 65 mmHg, and patients of the third cohort received protocolized care using the HPI.

**Results:**

Clinicians felt challenged to manage IOH in some patients, yet they reported sufficient knowledge and skills. HPI-software was considered useful and beneficial. No difference was found in incidence of IOH between cohorts. TWA was comparable between baseline and education cohort (0.15 mmHg [0.05–0.41] vs. 0.11 mmHg [0.02–0.37]), but was significantly lower in the HPI cohort (0.04 mmHg [0.00 to 0.11], p < 0.05 compared to both).

**Conclusions:**

Clinicians believed they had sufficient knowledge and skills, which could explain why no difference was found after the educational intervention. In the HPI cohort, IOH was significantly reduced compared to baseline, therefore HPI-software may help prevent IOH.

**Trial registration:**

ISRCTN 17,085,700 on May 9th, 2019.

**Supplementary Information:**

The online version contains supplementary material available at 10.1007/s10877-023-01097-z.

## Introduction

Intraoperative hypotension (IOH) is a frequent occurrence [1] and has been associated with various adverse outcomes [2–7]. The Hypotension Prediction Index (HPI) software was developed to predict hypotension prior to its occurrence, based on features of the arterial pressure waveform [8]. The HPI-software provides a unitless number between 0 and 100: the higher this number the more likely hypotension is about to occur and the shorter the time to the event [9]. The use of HPI-software has been shown to achieve a reduction in IOH in multiple settings [10–13]. However, in the largest trial to date, no difference was found between standard care and the protocolized use of the HPI-software [14]. Another more simplistic method to mitigate IOH might be by educating and mandating clinicians to keep MAP above a target threshold to achieve the same clinical endpoints. Hence, it may be questionable whether the use of HPI has any additive value. It is currently unclear what the role of clinicians’ beliefs and behavior is in this context. We therefore primarily explored clinicians’ beliefs around IOH itself, as well barriers to its treatment, *including* factors relating to the implementation of HPI technology into routine clinical practice. Secondarily, we aimed to assess whether an educational intervention including a mandated MAP target, or the protocolized use of HPI-software was associated with a reduction in IOH.

## Methods

This prospective cohort study was approved by the Ethics committee in both countries (UK: 18/YH/0185 and The Netherlands: 2018/00452) and the trial was registered on ISRCTN (ISRCTN 17,085,700 on May 9th, 2019). Written informed consent was obtained before the interviews from the participating clinicians and from patients prior to surgery.

**Fig. 1:**
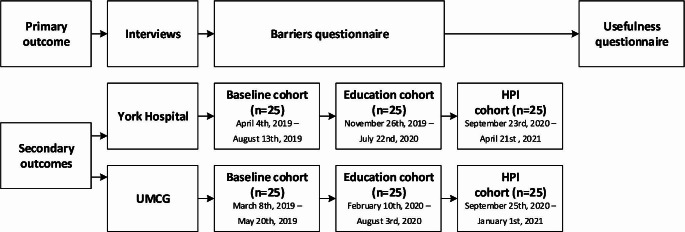
Study timeline

This manuscript was written in accordance with the STROBE statement [15]. This study consisted of two parts (Fig. 1 for a study timeline):

I) The exploration of beliefs around IOH, barriers to its treatment and factors relating to the implementation of HPI technology into routine clinical practice assessed by clinicians’ opinions derived from interviews and questionnaires.
II) Clinical outcomes regarding the effect of education and mandated MAP and the implementation of the HPI-software on IOH, which was assessed in three cohorts: a baseline cohort, an educational intervention combined with a mandated MAP cohort and a HPI cohort.

### Part I: behavioral aspects

The primary objective was to explore beliefs regarding IOH, barriers to its treatment, and factors relating to the implementation of HPI into clinical practice assessed by interviews and questionnaires. Semi-structured interviews with clinicians (anesthesiologist, residents, anesthetic nurses) were conducted in 2018 at both sites to explore behavioral factors in controlling hypotension. The Theoretical Domains Framework [16, 17], a comprehensive framework of factors influencing professional behavior was used to develop the interview (Supplementary Information).

Questionnaires were developed using the Influences on Patient Safety Behaviour Questionnaire and the aforementioned interviews used for input [18]. Questionnaires were distributed at both hospitals during the baseline and educational cohort. Clinicians were asked to provide feedback about factors that affect their management of intraoperative blood pressure, and about the perceived barriers (Barriers questionnaire; Supplementary Information). A second questionnaire was developed including items from the Implementation of medical devices questionnaire [19] to assess usefulness of HPI technology (Usefulness questionnaire, Supplementary Information) and was distributed to all staff who had used the HPI-software in practice.

### Part II: clinical application of HPI

The secondary objective was to assess if an educational intervention including a mandated MAP threshold or the protocolized use of the HPI algorithm was associated with a reduction in incidence of IOH, which was included to explore the effect of the interventions that we performed. Patients scheduled for elective major abdominal, orthopedic, head and neck or vascular surgery were considered eligible if the expected procedure duration was > 90 min and invasive blood pressure monitoring was required. Patients were excluded if there was a requirement for an intraoperative MAP < 65 mmHg or if they had a MAP < 65 mmHg at preoperative anesthetic evaluation. Patients with significant heart failure, intracardiac shunt, valvular abnormalities, cardiac arrhythmias, hepatic surgery, end-stage kidney failure, age < 18 years and refusal or inability to give informed consent were excluded. Patient inclusions took two years between 2019 and 2021.

### Intraoperative care

On arrival to theatre, an intravenous cannula was inserted, and an arterial cannula was placed for continuous blood pressure monitoring, and connected to an Acumen IQ transducer and HemoSphere monitoring platform (software version 1.1, Edwards Lifesciences, Irvine, USA). In the first two cohorts the arterial catheter was placed either before or after induction of general anesthesia. In the third cohort the arterial catheter was placed before induction of general anesthesia to obtain awake baseline values. No guidance on anesthetic technique was provided. After endotracheal intubation, the patients’ lungs were ventilated using the volume controlled mode with tidal volumes > 7 ml kg^− 1^.

#### Baseline cohort – standard institutional care

Twenty-five patients were included per cohort per site and received standard institutional care which included goal-directed fluid therapy to maintain a stroke volume variation (SVV) < 12% (14% in laparoscopic surgery; the treatment algorithm can be found in the Supplementary Information) and clinicians had access to all standard hemodynamic variables (blood pressure, cardiac index (CI), and SVV). Management of hypotension was at the discretion of the attending anesthesiologist.

#### Education cohort – maintain intraoperative MAP > 65 mmHg

After the baseline cohort, all clinicians were educated on the current evidence of the association between IOH and adverse outcomes, and it was emphasized that severity and duration of IOH are important determinants of organ injury and that a MAP > 65 mmHg should be maintained. A detailed description can be found in the Supplementary Information. Subsequently, twenty-five new patients per site were included in the education cohort. Clinicians were asked to rigorously maintain a MAP > 65 mmHg. No specific guidance was given on how to achieve this target. Clinicians had access to blood pressure, CI and SVV as in the previous cohort.

#### HPI cohort – Use of HPI-software with clinical guidance

**Fig. 2:**
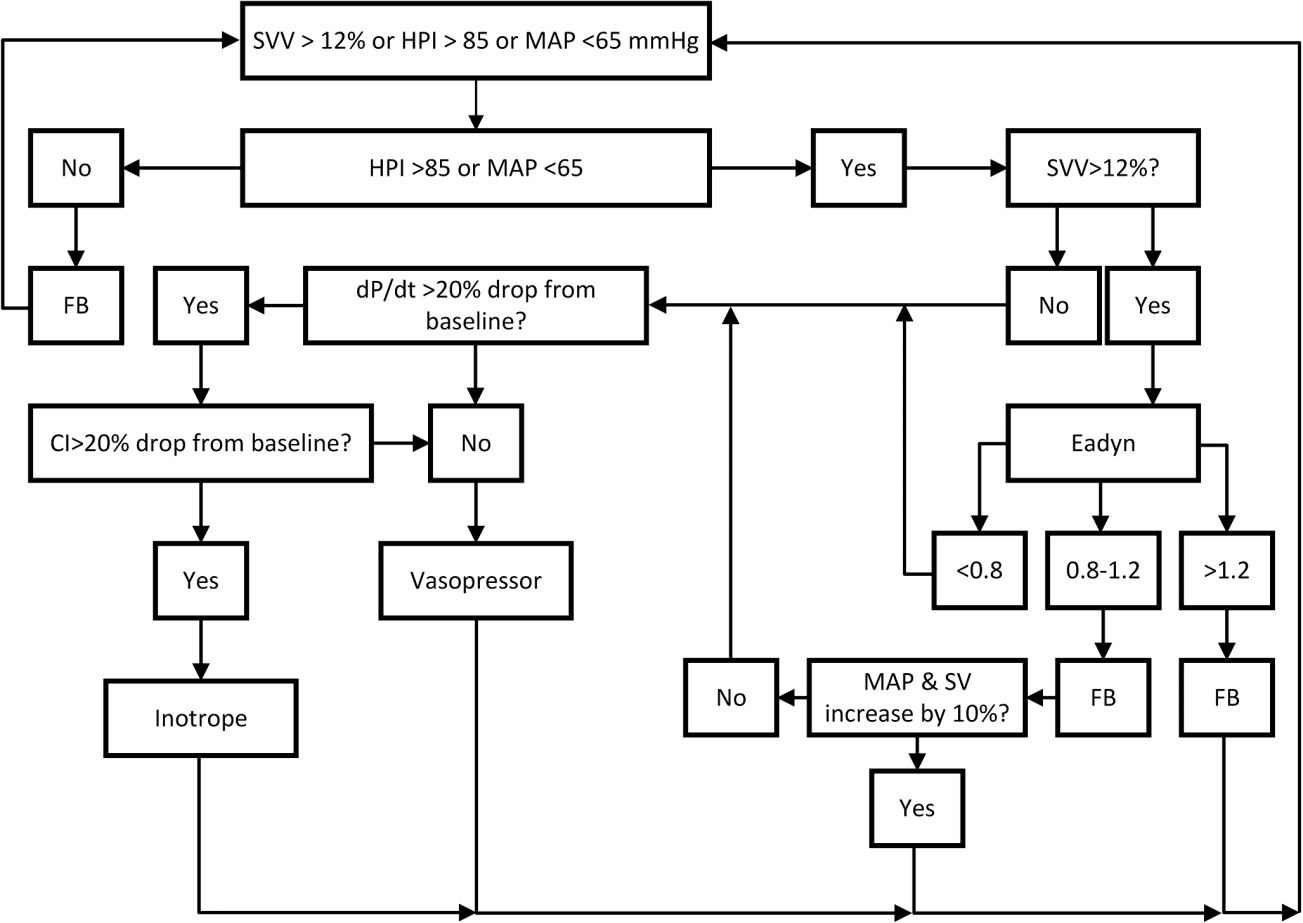
Treatment protocol of HPI cohort *SVV = stroke volume variation, HPI = Hypotension Prediction Index, MAP = mean arterial pressure, Ea*_*dyn*_ *= dynamic arterial elastance, SV = stroke volume, dP/dt = maximal change in arterial pressure over time, CI = cardiac index* The dynamic arterial elastance is the pulse pressure variation divided by the stroke volume variation and tells us if a patient who is fluid responsive will also increase their arterial pressure in response to fluid administration [20]. In the flow diagram above it means that patients with a high Ea_dyn_ (> 1.2) are likely to have an increase in arterial pressure and patients with a low Ea_dyn_ (< 0.8) are unlikely to have an increase in arterial pressure after fluid administration. In the grey zone (0.8–1.2) a fluid bolus should be given and the effect should be assessed. The dP/dt is a measure of cardiac contractility [21]. The SVV threshold was increased for patients undergoing laparoscopy to 14% [22–24].

After the education cohort, the same clinicians were educated on the HPI technology. Twenty-five new patients per site were recruited and clinicians were asked to keep MAP > 65 mmHg by using a treatment protocol with the HPI algorithm incorporated (Fig. 2). A member of the research team was present during the study procedures of all three cohorts to record interventions. The HPI-software provides HPI, dP/dt and Ea_dyn_ in a combined manner, therefore we were unable to incorporate a cohort where dP/dt and Ea_dyn_ were used without HPI.

After each study procedure, all hemodynamic data were downloaded from the HemoSphere and subsequently analyzed. IOH was expressed as the incidence, number of episodes with MAP < 65 mmHg for > 1 min per procedure, absolute duration and time-weighted average of IOH (TWA) from skin incision until skin closure. TWA is a combination of the severity and duration of hypotension, calculated by dividing the area under the threshold for defining hypotension by the duration of surgery. Potential over-treatment was assessed by analyzing the TWA of MAP > 100 mmHg. Furthermore, hemodynamic interventions were compared between cohorts.

### Statistical analysis

The results from the questionnaire are reported using the Likert Scale and expressed using mean scores and SD. Continuous variables are expressed as mean (standard deviation (SD)) or median [interquartile ranges (IQR)]. Normal distribution was assessed with the Shapiro-Wilk test. Continuous variables were compared between cohorts using ANOVA and Kruskal-Wallis tests. Categorical variables are presented as frequencies (percentages) and compared using the Chi-square test or the Fisher-exact test when indicated. Median differences were reported for clinical outcomes. Post-hoc tests (Chi-square test or the Fisher exact test) were conducted with a Bonferroni correction on all categorical variables. Instead of dividing all p-values by three, the p-value was multiplied by three and p < 0.05 remained statistically significant. For continuous variables, the Games-Howell corrected p-values were reported. The effect of confounders on the TWA was assessed by performing an exploratory regression analysis. Statistical analyses were conducted with R Studio (version 4.0.5, R Core Team, Vienna, Austria, 2020). A p-value < 0.05 was considered significant. Missing data were coded as missing, and no imputation was used.

### Sample size calculation

A model of theoretical sampling and saturation was used to determine the number of clinicians needed for the structured interviews. This ensured that we reflected key characteristics in the sample that were likely to introduce variety into the responses. Interviews were discontinued at a point where no new information was being generated i.e. we achieved saturation. It was estimated that a minimum of 5 clinicians would be needed from each site. Questionnaires were distributed to all available staff. In addition, including 50 patients per cohort would give an 80% power of detecting a 40% reduction in the incidence of MAP < 65 mmHg for more than 1 min from 65% in the baseline cohort [1], to the education cohort, and then a 60% reduction in the HPI cohort. By this sample size calculation we thus could investigate the quantitative, primary outcome conveniently and study the secondary quantitative outcome conveniently too.

## Results

### Part I: baseline beliefs amongst clinical staff of controlling hypotension during surgery

Twenty-seven clinicians were interviewed (anesthesiologist (n = 21), residents (n = 2) and anesthetic nurses (n = 4)), of which 16 were male. They considered managing hypotension as a priority and rarely forget to manage hypotension. Participants felt that some flexibility was required in the management of IOH. For example, younger patients may be better able to compensate physiologically, and short periods of hypotension were not considered too harmful by some. While clinicians’ knowledge about the clinical guidelines varied, they were generally confident in their own ability to manage hypotension adequately. However, they acknowledged that some patient factors (e.g., trauma, blood loss, renal or cardiovascular problems) could make it more difficult for them to manage IOH. Clinicians also commented that while their theatre team/colleagues were generally aware of hypotension, neither they, nor the surgeon, particularly encouraged or interfered with the management of IOH.

#### Perceived barriers to effective control of hypotension

Questionnaires based on the interviews were distributed to all departmental staff (n = 319) between July and October 2020. Eighty-four responses (26% response rate) were received from 75 doctors and 9 anesthetic nurses (Table 1). The first barrier was the belief that it was challenging in some patients to manage IOH (beliefs about capabilities mean 3.41, SD 1.75), that the surgeon and the surgical team did not necessarily encourage them to do this (social influences mean 2.88, SD 0.61), and that there should be flexibility in that having the same plan for every patient was not appropriate (action planning mean 2.81, SD 0.55). In contrast, the clinicians generally believed to have sufficient knowledge (mean 2.20, SD 0.67) and skills (mean 1.94, SD 0.60) to treat hypotension.

**Table 1:** Theoretical Domains Framework baseline questionnaire

Domain	Mean (SD) score N = 84
Beliefs about capabilities	3.41 (1.75)
Social influences	2.88 (0.61)
Action planning	2.81 (0.55)
Emotion	2.45 (0.70)
Cognitive, memory and decision making	2.37 (0.59)
Environmental context and resources	2.37 (0.76)
Motivation and goals	2.27 (0.72)
Knowledge	2.20 (0.67)
Beliefs about consequences	1.95 (0.59)
Skills	1.94 (0.60)
Professional role / identity	1.78 (0.69)

The scores range from 1 to 5, where 1 = strongly agree and 5 = strongly disagree. The mean score and SD is provided

#### Perceived usefulness and usability of predictive technology and how readily can it be integrated into routine clinical practice

Fourteen out of twenty clinicians that used HPI-software completed the questionnaire (reported on a scale from 1 (strongly disagree) to 5 (strongly agree) as mean, SD). They reported being happy to continue to use the HPI algorithm (4.14, SD 0.64), but they were somewhat neutral about being more likely to choose the version with HPI-software than standard hemodynamic monitoring (3.21, SD 1.08) (Supplementary Information). The views of clinicians about their experience of using HPI-software were slightly positive, finding it easy to use (4.00, SD 0.53) and useful (3.57, SD 0.99). However, at the same time they might not perceive a strong need to use it as they were not fully convinced that it would improve patient outcomes (2.93, SD 0.59), that it increased safety (3.29, SD 0.88), or is supported by evidence (3.35, SD 0.49). They were more or less neutral about the complexity of the treatment protocol (2.64, SD 0.74) and did not see that the technology changed their management of patients (3.07, SD 1.06).

### Part II: clinical outcomes

Eleven patients were excluded after enrolment in the baseline cohort (due to technical failure (n = 3) and logistical reasons (n = 8)). Twenty-two patients were excluded after enrolment in the education cohort (due to technical failure (n = 2), logistical reasons (n = 15), or at the discretion of the attending anesthesiologist (n = 5)) and twenty-seven patients were excluded from the third cohort after enrolment (due to logistical reasons (n = 20) or at the discretion of the attending anesthesiologist (n = 7)). A study flowchart can be found in the Supplementary Information. In total 150 patients were included (fifty per cohort) and patient characteristics, surgical and anesthetic details are shown in Table 2 and per center in the Supplementary Information.

**Table 2:** Patient characteristics, surgical and anesthetic characteristics

Cohort	Baseline (n = 50)	Education (n = 50)	HPI (n = 50)	P-value
Age (years)	67 (10)	68 (10)	67 (10)	0.969^1^
Male sex, n (%)	25 (50)	27 (54)	24 (48)	0.830^3^
BMI (kg m^− 2^)	27.5 [24.1 to 30.5]	26.5 [23.7 to 29.4]	27.0 [23.7 to 30.4]	0.754^2^
ASA				
I	2 (4)	1 (2)	0 (0)	0.095^4^
II	23 (46)	28 (56)	32 (64)	
III	25 (50)	18 (36)	18 (36)	
IV	0 (0)	3 (6)	0 (0)	
Preoperative SAP (mmHg)	141 (19)	138 (18)	141 (18)	0.664^1^
Preoperative DAP (mmHg)	73 (11)	78 (13)	75 (13)	0.130^1^
Preoperative MAP (mmHg)	96 (11)	98 (12)	98 (12)	0.644^1^
Type of surgery, n (%)				
Laparoscopic	14 (28)	24 (48)	24 (48)	0.064^3^
Open	36 (72)	26 (52)	26 (52)	
Specialty, n (%)				
Gastrointestinal	27 (54)	21 (42)	28 (56)	0.143^4^
Gynecology/urology	7 (14)	16 (32)	14 (28)	
Vascular	10 (20)	8 (16)	4 (8)	
Hepatobiliary	1 (2)	1 (2)	3 (6)	
Other	5 (10)	4 (8)	1 (2)	
Additional neuraxial analgesia, n (%)	31 (62)	30 (60)	40 (80)	0.063^3^
**Medical history**				
Smokers, n (%)	4 (8)	5 (10)	1 (2)	0.345^4^
Hypertension, n (%)	20 (40)	28 (56)	22 (44)	0.248^3^
Diabetes Mellitus, n (%)	7 (14)	5 (8)	5 (10)	0.851^3^
Myocardial infarction, n (%)	4 (8)	2 (4)	4 (8)	0.773^4^
Peripheral artery disease, n (%)	7 (14)	5 (10)	2 (4)	0.262^4^
Chronic obstructive pulmonary disease, n (%)	5 (10)	5 (10)	2 (4)	0.490^4^
**Medication**				
Beta-blocker, n (%)	7 (14)	10 (20)	10 (20)	0.666^3^
ACE-inhibitor, n (%)	7 (14)	12 (24)	9 (18)	0.434^3^
ATIIR blockers, n (%)	2 (4)	6 (12)	7 (14)	0.223^4^
Statin, n (%)	15 (30)	19 (38)	14 (28)	0.526^3^
**Intraoperative data**				
Anesthesia time (min)	284 [199 to 413]	271 [213 to 350]	312 [221 to 475]	0.450^2^
Surgery time (min)	221 [144 to 347]	220 [169 to 306]	249 [180 to 389]	0.457^2^
Blood loss (mL)	200 [100 to 500]	275 [100 to 625]	200 [100 to 463]	0.682^2^

^1^ANOVA ^2^Kruskal Wallis ^3^Chi-Square test ^4^Fisher’s Exact test. If a significant difference was found, a post-hoc test was performed. The reported p-value of the post-hoc test is the Games-Howell corrected p-value for continuous data and the actual p-value multiplied by three to account for multiple testing for categorical data (Bonferroni). ^#^ p < 0.05 between baseline and HPI cohort. Data are presented as mean (SD), median [interquartile ranges] or numbers (percentages)

BMI = Body mass Index, ASA = American Society of Anesthesiology Physical status, SAP = systolic arterial pressure, DAP = diastolic arterial pressure, MAP = mean arterial pressure, ACE = angiotensin converting enzyme, ATIIR = angiotensin II receptor

#### Intraoperative blood pressure

No difference was found in the incidence of IOH between cohorts (88% in the baseline cohort, versus 76% in the educational cohort and 72% in the HPI cohort, p = 0.127). The median number of hypotensive events (< 65 mmHg for > 1 min) per surgery was 3 [IQR 1 to 7] in the baseline cohort, which was not significantly different from that in the education cohort (3 [IQR 1 to 8], Table 3). The number of hypotensive events per surgery was 2 [IQR 0 to 4] in the HPI cohort, which was significantly lower than that in both the baseline (p = 0.016) and education cohort (p = 0.013). The surgical time spent in hypotension was 9.0 min [IQR 3.8 to 18.7] in the baseline cohort, 8.0 min [IQR 1.0 to 23.3] in the education cohort and 3.2 min [IQR 0.7 to 7.6] in the HPI cohort (HPI cohort vs. baseline p < 0.01 and HPI cohort vs. education p < 0.01). The TWA of MAP < 65 mmHg was 0.15 mmHg [IQR 0.05 to 0.41] in the baseline cohort, which was not significantly different from the education cohort (0.11 mmHg [IQR 0.02 to 0.37]). The TWA of MAP < 65 mmHg in the HPI cohort was 0.04 mmHg [IQR 0.00 to 0.11], which was significantly lower than the baseline (p = 0.012) and education cohort (p = 0.012). There was no increase in hypertension, defined as a TWA MAP > 100 mmHg, between any of the cohorts (Table 3). Blood pressure data per center can be found in the Supplementary Information. Multivariate analysis revealed that neither ASA classification (p = 0.270), nor baseline MAP (p = 0.151) affected the TWA of MAP < 65 mmHg. No difference was found in hemodynamic management between cohorts (Table 4), except significantly more patients received inotropic agents in the HPI cohort compared to baseline (64% vs. 34%, p = 0.015) and educational cohort (64% vs. 38%, p = 0.049).

**Table 3:** Intraoperative blood pressure

Cohort	Baseline (n = 50)	Education (n = 50)		HPI (n = 50)		P-value
Patients that suffered from at least one hypotensive event > 1 min, n (%)	44 (88)	38 (76)		36 (72)		0.127^1^
Hypotensive events > 1 min per procedure (n)	3 [1 to 7]	3 [1 to 8]		2 [0 to 4]		**0.019** ^**2#$**^
TWA of MAP < 65 mmHg (mmHg)	0.15 [0.05 to 0.41]	0.11 [0.02 to 0.37]		0.04 [0.00 to 0.11]		**0.001** ^**2#$**^
Surgery time of MAP < 65mmHg (min)	9.0 [3.8 to 18.7]	8.0 [1.0 to 23.3]		3.2 [0.7 to 7.6]		**0.002** ^**2#**&^
TWA of MAP > 100 mmHg (mmHg)	0.05 [0.00 to 0.33]	0.05 [0.00 to 0.20]		0.09 [0.04 to 0.48]		0.077^2^
**Median of differences** ^**3**^						
	**Baseline vs. Education**		**Education vs. HPI**		**Baseline vs. HPI**	
Hypotensive events > 1 min per procedure (n)	0.0 (95%CI: -1.0 to + 1.0)		1.0 (95%CI: 0.0 to + 3.0)		1.0 (95%CI: 0.0 to 2.0)	
TWA of MAP < 65 mmHg (mmHg)	0.01 (95%CI: -0.05 to 0.08)		0.06 (95%CI: 0.01 to 0.14)		0.08 (95%CI: 0.03 to 0.17)	
Surgery time of MAP < 65mmHg (min)	0.7 (95%CI: -3.3 to 5.0)		4.0 (95%CI: 0.7 to 9.7)		4.7 (95%CI: 1.7 to 8.0)	
TWA of MAP > 100 mmHg (mmHg)	0.00 (95%CI: -0.03 to 0.04)		-0.04 (95%CI: -8.54 to 0.00)		-0.04 (95%CI: -8.36 to 0.00)	

^1^Chi-Square test ^2^Kruskal Wallis ^3^Hodges Lehmann estimator. If a significant difference was found, a post-hoc test was performed. The reported p-value of the post-hoc test is the Games-Howell corrected p-value for continuous data. ^#^ p < 0.05 between baseline and HPI cohort, ^$^ p < 0.05 between education and HPI cohort, ^*^ p < 0.01 between baseline and HPI cohort, ^&^ p < 0.01 between education and HPI cohort. Data are presented as number (percentage) or median [interquartile ranges]

TWA = Time-weighted average, MAP = Mean arterial pressure

**Table 4:** Fluids, blood products and vasoactive agents administered by cohort

Cohort	Baseline (n = 50)	Education (n = 50)	HPI (n = 50)	P-value
**Fluids**				
Total volume of intraoperative fluids (mL)	2000 [1272 to 3212]	2000 [1500 to 2975]	2150 [1500 to 3000]	0.737^1^
Fluid bolus administered, n (%)	42 (84)	47 (84)	47 (84)	0.175^2^
Number of fluid boluses administered per patient	5 [3 to 10]	5 [3 to 7]	6 [3 to 9]	0.740^1^
**Blood products**				
Plasma, n (%)	2 (4)	0 (0)	2 (4)	0.547^2^
Total volume plasma (mL)	600 [500 to 700]	NA	600 [500 to 700]	> 0.99^1^
Packed red blood cells, n (%)	6 (12)	7 (14)	4 (8)	0.727^2^
Total volume packed red blood cells (mL)	640 [410 to 1020]	524 [402 to 646]	840 [539 to 2056]	0.452^1^
**Vasoactive agents**				
**Vasopressor agents, n (%)**	48 (96)	49 (98)	48 (96)	> 0.99^2^
Noradrenaline, n (%)	26 (52)	24 (48)	25 (50)	0.923^3^
Cumulative dose (μg)	1040 [516 to 1979]	1092 [753 to 1980]	704 [282 to 1480]	0.359^1^
Dosage (μg kg^− 1^ min^− 1^)	0.04 [0.03 to 0.09]	0.06 [0.04 to 0.08]	0.04 [0.02 to 0.08]	0.243^1^
Metaraminol, n (%)	23 (46)	25 (50)	23 (46)	0.899^3^
Cumulative dose (mg)	8.0 [5.0 to 14.6]	8.6 [5.8 to 11.3]	7.8 [5.5 to 10.2]	0.995^1^
Dosage (μg kg^− 1^ min^− 1^)	0.42 [0.20 to 0.59]	0.37 [0.26 to 0.46]	0.30 [0.18 to 0.39]	0.351^1^
Phenylephrine, n (%)	0 (0)	1 (2)	1 (2)	> 0.99^2^
Cumulative dose (μg)	NA	150^%^	50^%^	> 0.99^1^
**Inotropic agents, n (%)**	17 (34)	19 (38)	32 (64)	**0.005** ^**3#$**^
Ephedrine, n (%)	15 (30)	19 (38)	31 (62)	**0.004** ^**3&**^
Cumulative dose (mg)	9.0 [6.0 to 13.5]	6.0 [5.0 to 11.0]	12.0 [6.0 to 18.0]	0.072^1^
Dobutamine, n (%)	2 (4)	0 (0)	7 (14)	**0.011** ^**2$**^
Cumulative dose (mg)	33.3 [28.4 to 38.1]	NA	15.0 [8.7 to 37.4]	0.380^1^
Dosage (μg kg^− 1^ min^− 1^)	1.85 [1.79 to 1.90]	NA	1.24 [0.79 to 1.64]	0.242^1^

^1^Kruskal Wallis ^2^Fisher’s Exact test ^3^Chi-Square test. If a significant difference was found, a post-hoc test was performed. The reported p-value is the actual p-value multiplied by three to account for multiple testing for categorical data (Bonferroni). ^#^ p < 0.05 between baseline and HPI cohort, ^$^ p < 0.05 between education and HPI cohort, ^&^ p < 0.01 between education and HPI cohort. ^%^This intervention was received by only one patient per cohort. Data are presented as median [interquartile ranges] or numbers (percentages)

## Discussion

We aimed to explore beliefs regarding IOH and barriers to its treatment, and found that clinicians considered managing hypotension important, felt they had the knowledge and skills to effectively treat hypotension, but lacked capabilities in specific situations. No difference was found in the incidence of IOH between the three cohorts, and no decrease in hypotensive events or TWA of IOH was found in the educational cohort compared to baseline. In contrast the HPI cohort showed less hypotensive events and a lower TWA of hypotension compared to both other groups. The users found the HPI-software easy to use, useful, and the experience of using the technology was positive, however, clinicians were skeptical that it would improve patient outcomes.

Clinicians believed that they had sufficient knowledge and skills to treat IOH adequately could explain why an educational intervention *and* asking clinicians to keep the MAP > 65 mmHg was not associated with a reduction in IOH. This however is at odds with the main barrier to managing hypotension identified, which centered around beliefs about capabilities, that for example for some patients managing hypotension is challenging. Additional barriers identified included ‘action planning’ (the identification of patients in which blood pressure management is a key priority) and ‘social influences’ (clinicians were not encouraged to manage hypotension by the wider team). We do not suggest that the surgeon is responsible for managing blood pressure, but team work, collaboration and mutual awareness regarding the importance of IOH may make the task of managing it easier and more effective. Clinicians were happy using the HPI-software, finding it easy to use and useful. However, they were somewhat neutral about being more likely to choose the version with HPI-software over standard hemodynamic monitoring, and were skeptical that it would improve patient outcomes, increase safety and was supported by research evidence, since little evidence regarding the use of HPI-software and outcomes had been described at the time the study was performed [25, 26]. Nevertheless, IOH decreased in the HPI cohort compared to the baseline cohort, despite an already low TWA of hypotension in the baseline cohort compared to other studies [11, 27]. Since all studied populations consisted of patients undergoing major surgery, we believe that the difference in TWA of hypotension may be secondary to an already high blood pressure management awareness, which is supported by the interviews we conducted. It remains to be elucidated whether such a (further) reduction in IOH would lead to an improvement of postoperative outcomes, which is a topic beyond the scope of this paper. Clinicians did not feel as if the HPI-software changed their management of patients, even though inotropes were used more often in the HPI cohort (in accordance with the treatment algorithm) – indicating the contrary. For vasopressors, there was no difference in their use between cohorts, mostly as they were already used in almost all patients. Concerningly, there was a belief that short periods of hypotension are not harmful, which is not consistent with current evidence [6, 28, 29].

Multiple RCT’s have found a reduction in hypotension when the HPI-software was used compared to standard care [10–13] and a recent registry study showed IOH was low when using the HPI-software [30]. However, one RCT did not show a reduction in IOH by the protocolized use of the HPI-software (median TWA 0.14 mmHg [IQR 0.03–0.37] vs. 0.14 mmHg [IQR 0.03–0.39]) [14]. A possible explanation for this contradicting result was the complexity of a treatment protocol based on HPI, leading to low protocol adherence. Only 45% of HPI warnings issued an intervention. Therefore, poor compliance, and an overly complicated protocol may have been the cause of the negative result. For example, we previously demonstrated that a high protocol compliance is critically important for truly optimizing postoperative outcome in implementing a goal-directed therapy protocol [31]. In this study the clinicians were neutral about the complexity of the protocol, although the protocol was similar to that of the previous study. A key issue for the success of this type of technology will be adequate training and guidance during implementation.

Criticism regarding HPI-software validation has emerged after completion of this study [32]. Therefore, predictive abilities of linearly extrapolated MAP and single MAP values with a maximum time to event of 5 min were assessed recently [33], which suggests that the predictive abilities are comparable to the area under the curves (AUC) reported in the original validation paper of HPI [9]. However, it must be noted that extrapolation will always lead to very high or low values of MAP at a longer term, and that single MAP values and extrapolation of MAP is prone to incorporating artefacts. Comparable AUC’s for MAP and HPI were additionally found in a direct comparison of MAP and HPI in liver transplant recipients [34]. Moreover, a strong cross-correlation was found between MAP and HPI (–0.91 ± 0.04) [35]. Therefore, setting an alarm at a MAP value of e.g. 70 mmHg was therefore encouraged instead of using complex algorithms [36]. This approach has not currently been shown to reduce intraoperative hypotension. Also, it was apparent in our study that in our second cohort without HPI-software, IOH was still not reduced compared to baseline, despite education and mandating normotension, emphasizing the need to set an actual alarm. Given that MAP alarms have been available for many decades it is uncertain if this approach will work, and mandated blood pressure targets, as in this study and others, have not been associated with a reduction in hypotension compared to using HPI.

Despite the strong associative evidence between IOH and adverse outcomes [2–7, 37], a clear causal relationship has not been established yet, since the number of studies targeting intraoperative blood pressure management are limited and they remain inconclusive [38, 39]. Until then, we believe it is best to follow the consensus guidelines and avoid intraoperative hypotension (MAP > 60–70 mmHg) [2]. Despite this, the first effects of HPI algorithm on outcome have recently been published [25, 26].

There are some limitations to our study. First, the study was conducted amidst the pandemic, and this could have negatively impacted the response rate on the questionnaires and caused significant logistical challenges during the education and HPI cohort. Second, this was a sequential study where time and training could have influenced our results. Third, the design might have induced a selection bias, although we did not find relevant differences in patient demographics, surgical and anesthetic characteristics between the cohorts. We did find a difference in baseline TWA of hypotension between both centers (Supplementary Information), yet the decrease in TWA of hypotension in the HPI cohort was present at both institutions. Both are academic centers, but are located in different countries, therefore differences in type of procedures, type of patients and anesthetic management are inevitable. Fourth, a subgroup analysis of the questionnaires per profession would have been interesting, but the responses from non-anesthesiologist were limited, therefore we refrained from performing this analysis. Fifth, we focused on addressing both behavioral aspects and changes in IOH, but we did not investigate postoperative outcomes.

## Conclusion

Our exploratory analysis showed that clinicians considered treating IOH important, were confident in their skills and knowledge, but were lacking capabilities in specific situations to effectively treat IOH. Therefore, it was not surprising that IOH was not reduced in the educational cohort with a mandated blood pressure target. In the HPI cohort, the number of hypotensive events, duration and TWA of IOH were reduced compared to baseline. The limited number of clinicians that used the HPI-software considered it useful, easy to use, safe, and beneficial. Our results support the necessity for an early alarm function such as the HPI software in order to prevent IOH. Superiority of the HPI-software over an alarm on a vital signs monitor set at a MAP of 70 mmHg or a linearly extrapolated MAP needs to be assessed.

### Electronic supplementary material

Below is the link to the electronic supplementary material.

Supplementary Material 1

## Declarations

### Presentation

Preliminary data were presented at Euroanaesthesia 2021 on December 18^th^.
