# Supplementary material for: Impact of clinicians’ behavior, an educational intervention with mandated blood pressure and the hypotension prediction index software on intraoperative hypotension: a mixed methods study
Source: J Clin Monit Comput. 2023 Dec 19;38(2):325–35. doi: 10.1007/s10877-023-01097-z (PMC10995090; doi:10.1007/s10877-023-01097-z)
Supplement: Supplementary file 1 — Supplementary Material 1 [file 10877_2023_1097_MOESM1_ESM.docx]

**Supplementary Information**

**Interview**

We are conducting interviews as we wish to understand the beliefs amongst clinical staff of the costs and benefits of controlling hypotension during surgery following initial training.

1. To what extent do you see managing hypotension perioperatively as a priority? Why?

2. How do you manage hypotension and how have you learnt to do this?

Prompt: Do you refer to any guidance/ training/ literature?

3. In what circumstances would you be less concerned and/or more concerned to manage hypotension?

4. What are the short- and long-term benefits for the patient/ you/ the team or service of managing hypotension?

5. Are there any negative consequences?

6. Do your colleagues (other anesthesiologist) strive to manage hypotension?

7. Thinking about other people you work with in theatres - how you interested are they in managing hypotension?

8. Do you feel you have the knowledge and skills you need to manage hypotension?

9. How difficult is it to manage hypotension? Why is this?

10. What tools or equipment do you have to help you manage hypotension? Is there anything else you need?

11. Is managing hypotension something that you are measured on or encouraged to do by anyone else?

12. Are there situations in which it is difficult or impossible to manage hypotension? How does this make you feel?

13. Is managing hypotension something you do automatically, or do you sometimes forget?

14. Is managing hypotension an interesting or satisfying part of the job?

15. To what extent is the ability to manage hypotension a sign that you are good at your job?

16. Is there anything else that you would like to add?

**Barriers questionnaire**

| a) Name of hospital: |  |
| --- | --- |
| b) Specialty/work area: |  |
| c) Profession (please circle) | Staff/consultant  Resident  Anesthetic nurse |
| d) We are interested in your knowledge and use of the ‘Perioperative Quality Initiative consensus statement on the physiology of arterial blood pressure control in perioperative medicine’ (Ackland, Brudney, Mauruzo et al, 2019, British Journal of Anaesthesia, Volume 122, pages 542 – 551) (<https://bjanaesthesia.org/article/S0007-0912(19)30047-9/fulltext> )   1. Please indicate (circle) if you are familiar with the details of the ‘Perioperative Quality Initiative consensus statement on the physiology of arterial blood pressure control in perioperative medicine’:   No  Yes   1. **Being completely honest**, how much does your practice adhere to the above consensus statement on a scale of 1-5 (please circle):   Not at all  Very much    1 2 3 4 5 | |
| e) Please add any comments about what factors would help you to manage hypotension perioperatively in patients over 60 years of age undergoing major surgery. | |

Below you are asked about a range of things that might affect you being able to manage hypotension perioperatively in patients over 60 years of age undergoing major surgery.

Please indicate your level of agreement with each statement by circling the relevant number on the scale**.**

| Question list | End statement | Strongly  Agree | Agree | Neither agree nor disagree | Disagree | Strongly  disagree |
| --- | --- | --- | --- | --- | --- | --- |
| Other priorities get in the way of me being able to... | manage intra-operative blood pressure to a MAP of 65 or above for patients over 60 years of age undergoing major surgery | 1 | 2 | 3 | 4 | 5 |
| There is not a good enough monitoring system in place to… |  | 1 | 2 | 3 | 4 | 5 |
| Sometimes I find it frustrating when trying to… |  | 1 | 2 | 3 | 4 | 5 |
| I know what I need to do to … |  | 1 | 2 | 3 | 4 | 5 |
| It is not clear what the evidence is to… |  | 1 | 2 | 3 | 4 | 5 |
| I am confident in the evidence underpinning guidelines about how and when to… |  | 1 | 2 | 3 | 4 | 5 |
| I have developed the skills I need to… |  | 1 | 2 | 3 | 4 | 5 |
| It is a very important part of my role to… |  | 1 | 2 | 3 | 4 | 5 |
| I am confident in my ability to… |  | 1 | 2 | 3 | 4 | 5 |
| It is very important for patient outcomes to… |  | 1 | 2 | 3 | 4 | 5 |
| I don’t even think about it -it is automatic to just… |  | 1 | 2 | 3 | 4 | 5 |
| I have the necessary resources (e.g., correct/enough equipment, staff, drugs etc.) to… |  | 1 | 2 | 3 | 4 | 5 |
| Surgeons encourage me to prioritize to… |  | 1 | 2 | 3 | 4 | 5 |
| I get a lot of job satisfaction when I… |  | 1 | 2 | 3 | 4 | 5 |
| I have a clear plan for each patient about how to… |  | 1 | 2 | 3 | 4 | 5 |
| Patients do better post-op if the time they are hypotensive is minimized so it is important to… |  | 1 | 2 | 3 | 4 | 5 |
| There is a need to allow for individual patient variation when I… |  | 1 | 2 | 3 | 4 | 5 |
| Patient factors can make it difficult to… |  | 1 | 2 | 3 | 4 | 5 |
| It will be bad for the patient if I do not... |  | 1 | 2 | 3 | 4 | 5 |
| There are justifiable reasons for why I often decide not to… |  | 1 | 2 | 3 | 4 | 5 |
| The teams I work with would support me to … |  | 1 | 2 | 3 | 4 | 5 |
| I often don’t have all the clinical information I need to be able to… |  | 1 | 2 | 3 | 4 | 5 |
| I feel a sense of pride when I do a good job of being able to… |  | 1 | 2 | 3 | 4 | 5 |

| Any other factors? Please write in this section. |
| --- |

**Goal-directed fluid therapy algorithm**

**
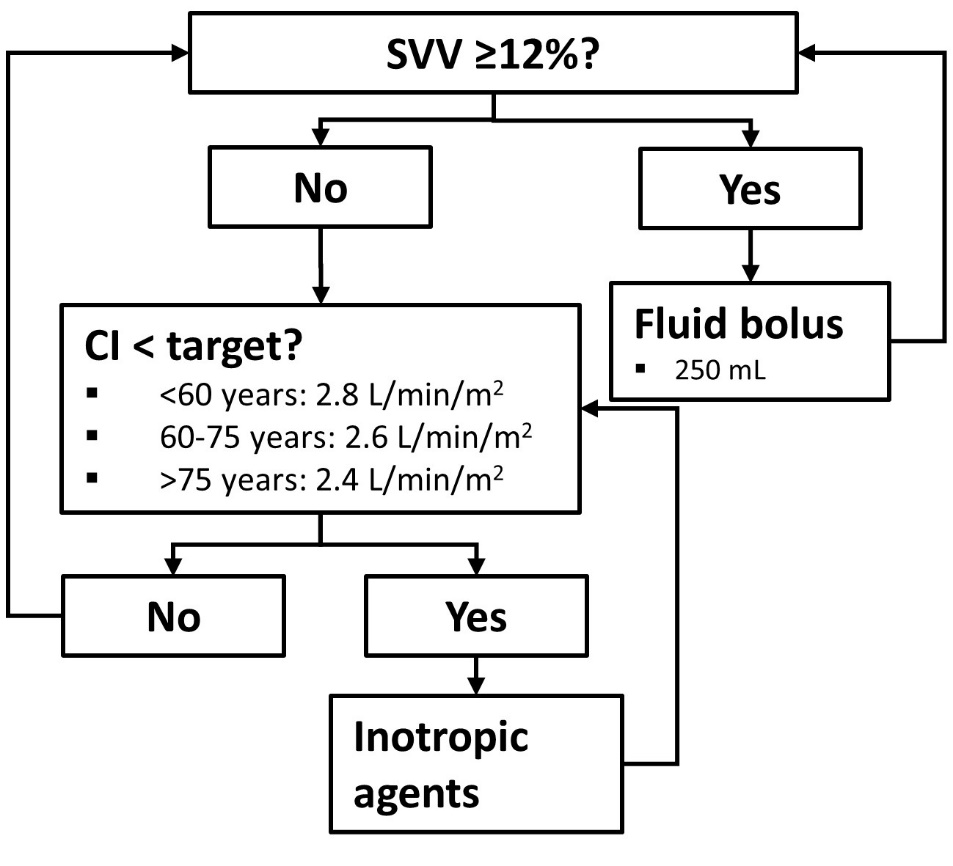
**

**Detailed description of educational intervention in educational cohort**

A presentation was provided for each staff member participating in this cohort. The presentation consisted of an overview of the latest perioperative quality initiative consensus guideline on intraoperative blood pressure (BJA 2019; 122:563-574). Furthermore, the association between IOH and adverse outcomes was discussed and it was emphasized that injury was a function of hypotension severity and duration. GDT is standard care in both institutions and the clinicians were asked to maintain a MAP > 65 mmHg using GDFT including SVV and CI. The GDFT algorithm is shown above.

**Flowchart participants**


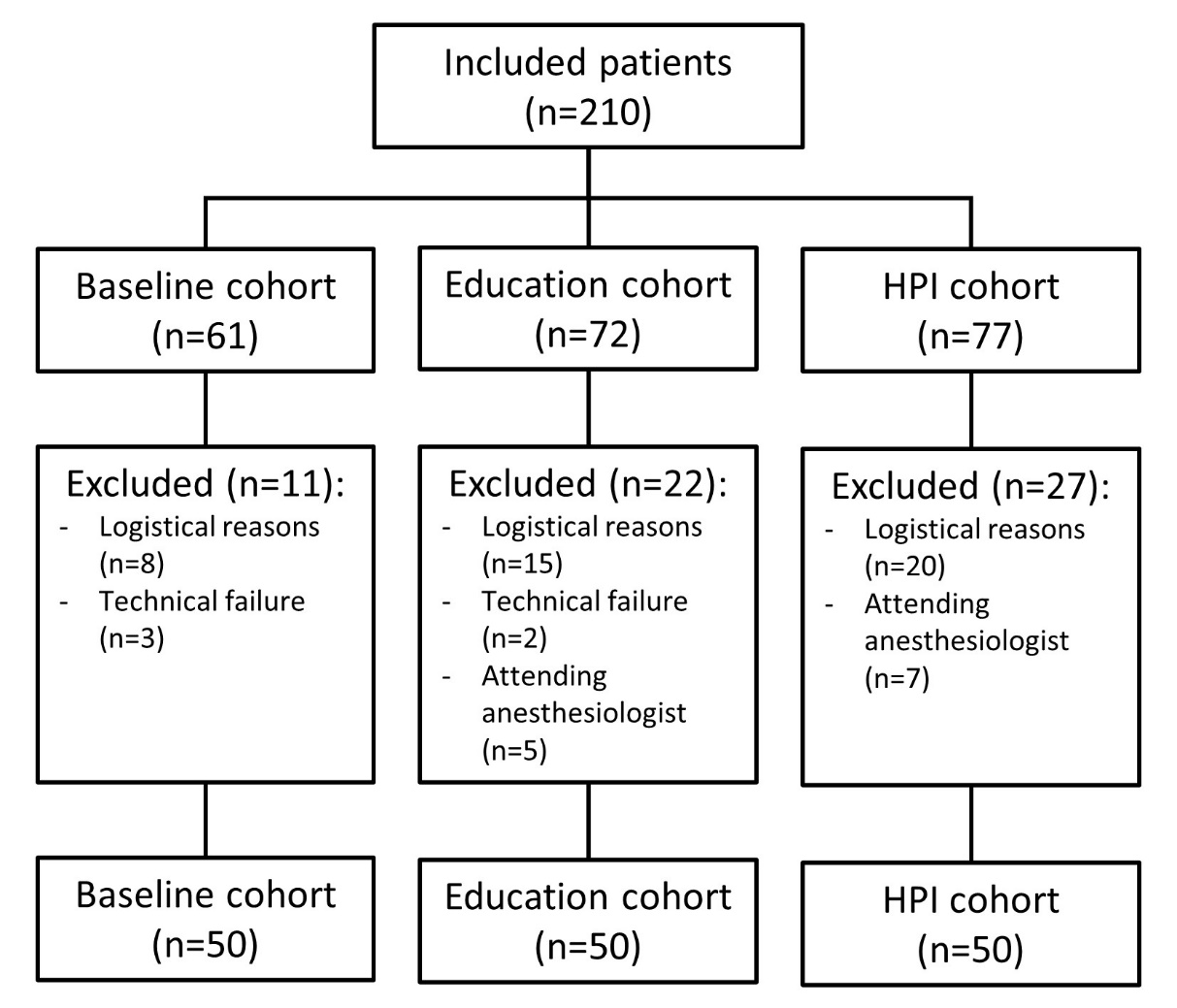


**Usefulness and usability of Acumen IQ Questionnaire**

| **Item** | **Mean (SD)**  **score (N=14)** |
| --- | --- |
| I would be happy to continue to use Acumen IQ with HPI with patients requiring invasive arterial monitoring | 4.14 (0.64) |
| The Acumen IQ with HPI equipment is easy to use | 4.00 (0.53) |
| Overall, I am satisfied with the Acumen IQ equipment | 3.64 (0.49) |
| The Acumen IQ with HPI equipment is useful | 3.57 (0.99) |
| Use of the Acumen IQ with HPI equipment has obvious benefits | 3.50 (0.74) |
| Use of the Acumen IQ with HPI equipment is supported by research evidence | 3.35 (0.49) |
| Use of the Acumen IQ with HPI equipment enhances the safety of anesthesia | 3.29 (0.88) |
| The protocol for the Acumen IQ with HPI equipment is easy to follow | 3.29 (0.72) |
| I would choose to use the Acumen IQ with HPI over the standard Acumen equipment | 3.21 (1.08) |
| The Acumen IQ with HPI equipment changes the way I manage a patient during anesthesia | 3.07 (1.06) |
| Use of the Acumen IQ with HPI equipment makes anesthesia more efficient | 3.07 (0.88) |
| The Acumen IQ with HPI equipment changes the way in which an anesthetic is delivered | 3.00 (0.96) |
| The use of the Acumen IQ with HPI equipment will improve patient outcomes post-operatively, e.g., speed of recovery, acute kidney injury, rate of readmission | 2.93 (0.59) |
| The Acumen IQ with HPI equipment is very different from the standard invasive monitoring equipment | 2.64 (1.06) |
| The protocol for the Acumen IQ with HPI equipment is too complicated | 2.64 (0.74) |
| The Acumen IQ with HPI equipment is complex | 2.63 (0.91) |
| Use of the Acumen IQ with HPI equipment requires a large amount of conscious mental effort | 2.63 (0.99) |
| Use of the Acumen IQ with HPI equipment involves a large amount of confusion | 2.14 (0.74) |
| Use of the Acumen IQ with HPI equipment involves a large amount of frustration | 2.07 (0.70) |
| Use of the Acumen IQ with HPI equipment involves a large amount of anxiety | 1.93 (0.70) |
| The scores range from 1 to 5, where 1 = strongly disagree and 5 = strongly agree. The mean score is provided. | |

| **Patient demographics per center** | | | | | | | | |
| --- | --- | --- | --- | --- | --- | --- | --- | --- |
|  | **University Medical Center Groningen (n=25)** | | | | **York Hospital (n=25)** | | | |
| **Cohort** | **Baseline** | **Education** | **HPI** | **P-value** | **Baseline** | **Education** | **HPI** | **P-value** |
| Age (years) | 65±11 | 66±10 | 66±10 | 0.989^1^ | 69±10 | 70±9 | 68±9 | 0.888^1^ |
| Male sex, n (%) | 10(40) | 13(52) | 10(40) | 0.615^3^ | 15(60) | 14(56) | 14(56) | 0.947^3^ |
| BMI (kg m^-2^) | 25.6±5.3 | 25.3±3.7 | 27.1±5.0 | 0.359^1^ | 28.7 [26.8 to 30.9] | 27.3 [25.2 to 31.2] | 27.7 [24.10 to 30.7] | 0.837^2^ |
| ASA  I  II  III  IV | 1 (4)  13 (52)  11 (44)  0 (0) | 1 (4)  15 (60)  8 (32)  1 (4) | 0 (0)  19 (76)  6 (24)  0 (0) | 0.386^4^ | 1 (4)  10 (40)  14 (56)  0 (0) | 0 (0)  13 (52)  10 (40)  2 (8) | 0 (0)  13 (52)  12 (48)  0 (0) | 0.389^4^ |
| Preoperative SAP (mmHg) | 145±17 | 137±17 | 138±16 | 0.176^1^ | 137±21 | 140±19 | 144±21 | 0.460^1^ |
| Preoperative DAP (mmHg) | 75±11 | 77±12 | 76±12 | 0.704^1^ | 72±10 | 79±14 | 75±14 | 0.144^1^ |
| Preoperative MAP (mmHg) | 98±11 | 97±9 | 97±11 | 0.926^1^ | 94±12 | 99±14 | 98±13 | 0.347^1^ |
| Type of surgery, n (%)  Laparoscopic  Open | 0 (0)  25 (100) | 4 (16)  21 (84) | 1 (4)  24 (96) | 0.119^4^ | 14 (56)  11 (44) | 20 (80)  5 (20) | 23 (92)  2 (8) | **0.015^4#^** |
| Specialism, n (%)  Gastrointestinal  Gynaecology/urology  Vascular  Hepatobiliary  Other | 7 (28)  6 (24)  7 (28)  1 (4)  4 (16) | 3 (12)  11 (44)  7 (28)  1 (4)  3 (12) | 4 (16)  13 (52)  4 (16)  3 (12)  1 (4) | 0.357^4^ | 20 (80)  1 (4)  3 (12)  0 (0)  1 (4) | 18 (72)  5 (20)  1 (4)  0 (0)  1 (4) | 24 (96)  1 (4)  0 (0)  0 (0)  0 (0) | **0.101^4^** |
| Additional neuraxial analgesia, n (%) | 17 (68) | 15 (60) | 16 (64) | 0.841^4^ | 14 (56) | 15 (60) | 24 (96) | **0.001^4*&^** |
| **Medical history** | | | | | | | | |
| Smokers, n (%) | 4 (16) | 2 (8) | 1 (4) | 0.487^4^ | 0 (0) | 3 (12) | 0 (0) | 0.102^4^ |
| Hypertension, n (%) | 8 (32) | 13 (52) | 10 (40) | 0.352^3^ | 12 (48) | 15 (60) | 12 (48) | 0.618^3^ |
| Diabetes Mellitus, n (%) | 2 (8) | 3 (12) | 1 (4) | 0.866^4^ | 5 (20) | 2 (8) | 4 (16) | 0.602^4^ |
| Myocardial infarction, n (%) | 3 (12) | 1 (4) | 3 (12) | 0.687^4^ | 1 (4) | 1 (4) | 1 (4) | >0.99^4^ |
| Peripheral artery disease, n (%) | 5 (20) | 4 (16) | 2 (8) | 0.602^4^ | 2 (8) | 1 (4) | 0 (0) | 0.769^4^ |
| Chronic obstructive pulmonary disease, n (%) | 5 (20) | 4 (16) | 2 (8) | 0.602^4^ | 0 (0) | 1 (4) | 0 (0) | >0.99^4^ |
| **Medication** | | | | | | | | |
| Beta-blocker, n (%) | 3 (12) | 7 (28) | 7 (28) | 0.330^4^ | 4 (16) | 3 (12) | 3 (12) | >0.99^4^ |
| ACE-inhibitor, n (%) | 3 (12) | 6 (24) | 6 (24) | 0.519^4^ | 4 (16) | 6 (24) | 3 (12) | 0.645^4^ |
| ATIIR blockers, n (%) | 0 (0) | 4 (16) | 3 (12) | 0.150^4^ | 2 (8) | 2 (8) | 4 (16) | 0.718^4^ |
| Statin, n (%) | 7 (28) | 9 (36) | 5 (20) | 0.452^3^ | 8 (32) | 10 (40) | 9 (36) | 0.841^3^ |
| **Intraoperative data** | | | | | | | | |
| Anesthesia time (min) | 271 [186 to 348] | 244 [211 to 373] | 229 [156 to 325] | 0.517^2^ | 301 [213 to 440] | 278 [232 to 338] | 425 [311 to 520] | **0.007^2&^** |
| Surgery time (min) | 220 [139 to 309] | 215 [166 to 317] | 186 [126 to 270] | 0.582^2^ | 241 [154 to 351] | 224 [174 to 291]^3^ | 373 [236 to 455]^2^ | **0.007^2&^** |
| Blood loss (mL) | 300 [100 to 1000] | 400 [200 to 900] | 200 [125 to 700] | 0.635^2^ | 200 [100 to 365] | 100 [100 to 300] | 100 [100 to 350] | 0.649^2^ |
| *^1^ANOVA ^2^Kruskal Wallis ^3^Chi-Square test ^4^Fisher’s Exact test. If a significant difference was found, a post-hoc test was performed. The reported p-value of the post-hoc test is the Games-Howell corrected p-value for continuous data and the actual p-value multiplied by three to account for multiple testing for categorical data (Bonferroni). ^#^ p<0.05 between baseline and HPI cohort,^*^ p<0.01 between baseline and HPI cohort, ^&^ p<0.01 between education and HPI cohort. Data are presented as mean (sd), median [interquartile ranges] or numbers (percentages).*  BMI = Body mass Index, ASA = American Society of Anesthesiology Physical status, SAP = systolic arterial pressure, DAP = diastolic arterial pressure, MAP = mean arterial pressure, ACE = angiotensin converting enzyme, ATIIR = angiotensin II receptor | | | | | | | | |

**Intraoperative blood pressures per center**

|  | **University Medical Center Groningen** | | | | **York Hospital** | | | |
| --- | --- | --- | --- | --- | --- | --- | --- | --- |
|  | **Baseline** | **Education** | **HPI** | **P-value** | **Baseline** | **Education** | **HPI** | **P-value** |
| Patients that suffered from at least one hypotensive event, n (%) | 24 (96) | 23 (92) | 21 (84) | 0.487^1^ | 20 (80) | 15 (60) | 15 (60) | 0.223^2^ |
| Hypotensive events >1 min per procedure (n) | 4 [2 to 9] | 7 [2 to 8] | 2 [1 to 4] | **0.020^3$^** | 2 [1 to 4] | 1 [0 to 3] | 1 [0 to 2] | 0.088^3^ |
| TWA of MAP <65mmHg (mmHg) | 0.26 [0.06 to 0.88] | 0.26 [0.15 to 0.71] | 0.07 [0.02 to 0.23] | **0.012^3#$^** | 0.08 [0.03 to 0.18] | 0.06 [0.00 to 0.18] | 0.02 [0.00 to 0.07] | **0.024^3#^** |
| Surgery time of MAP <65mmHg (min) | 13.3 [5.0 to 39.7] | 19.7 [5.7 to 26.7] | 6.7 [2.7 to 10.3] | **0.007^3#$^** | 6.3 [3.0 to 10.7] | 3.3 [0.7 to 8.7] | 1.7 [0.3 to 4.0] | **0.030^3^** |
| TWA of MAP >100mmHg (mmHg) | 0.13 [0.01 to 0.47] | 0.07 [0.03 to 0.21] | 0.12 [0.06 to 0.63] | 0.230^3^ | 0.01 [0.00 to 0.18] | 0.01 [0.00 to 0.12] | 0.07 [0.01 to 0.22] | 0.188^3^ |
| *^1^Fisher’s Exact test ^2^Chi-Square test ^3^Kruskal Wallis. If a significant difference was found, a post-hoc test was performed. The reported p-value of the post-hoc test is the Games-Howell corrected p-value for continuous data. ^#^ p<0.05 between baseline and HPI cohort, ^$^ p<0.05 between education and HPI cohort, ^*^ p<0.01 between baseline and HPI cohort, ^&^ p<0.01 between education and HPI cohort. Data are presented as number (percentage) or median [interquartile ranges].*  TWA = Time-weighted average, MAP = Mean arterial pressure | | | | | | | | |
